# Evaluating the quality and reliability of oncologic breast reconstruction videos on youtube

**DOI:** 10.1016/j.jpra.2024.10.006

**Published:** 2024-10-11

**Authors:** Daniel Soroudi, Israel Falade, Nina Mehta, Alexis Gursky, Raymond Yin, Siyou Song, Esther Kim

**Affiliations:** a School of Medicine, University of California San Francisco, San Francisco, CA, USA; b Chapel Hill School of Medicine, University of North Carolina, Chapel Hill, NC, USA; c Norton College of Medicine, SUNY Upstate Medical University, Syracuse, NY, USA; d Department of Surgery, Division of Plastic and Reconstructive Surgery, University of California San Francisco, San Francisco, CA, USA

**Keywords:** Oncoplastic breast reconstruction, Social media, Youtube, Plastic surgery, Patient education

## Abstract

Oncologic breast reconstruction (OBR) is a complex process that requires consideration of multiple factors, including chemoradiation, extent of cancer treatment, and surgical approach. Patients often feel uncertain about the numerous surgical options and may turn to popular social media platforms like YouTube for information. Thus, this study aims to assess the quality and reliability of YouTube videos related to OBR.

We conducted a retrospective, cross-sectional analysis of YouTube videos related to OBR. Search terms were obtained from plasticsurgery.org and Google Trends. The first ten videos for each search term were analyzed. Videos were categorized by source and subject matter and independently reviewed by three evaluators using the DISCERN scale.

This study examined 172 YouTube videos. Five video source categories were identified: Health Care Administrations, Physicians, Non-physician Providers, News Organizations, and Patients. Health Care Administration accounts received the highest overall DISCERN score of 3.6 ± 0.51, followed by Physicians at 2.98 ± 0.93, with News Organization accounts scoring the lowest at 2.22 ± 0.60 (*p* < 0.001). Videos from academic sources (Physicians and Health Care Administrators) had higher DISCERN scores compared to non-academic sources (News Organizations and Patients), 2.98 ± 0.95 versus 2.28 ± 0.77, respectively (*p* < 0.001).

Our findings indicate that videos from academic sources generally exhibit higher DISCERN scores, pointing to a higher content quality and reliability standard. Given the increasing reliance on YouTube for healthcare information, our study underscores the need for healthcare professionals to engage more actively in content creation and dissemination.

## Introduction

Approximately 240,000 new cases of breast cancer are diagnosed in women per year in the United States.^1^ Oncologic breast reconstruction (OBR) is a complex process that requires consideration of multiple factors, including chemoradiation, extent of cancer treatment, and surgical approach. Yet, patients may feel dissatisfied with the information they receive before surgery.^2^, ^3^

Many patients are turning to the Internet for health information, often well ahead of an initial consultation with a physician.^4^ Among the various online platforms, YouTube has emerged as one of the most popular choices, with a user base exceeding 2.68 billion.^5^ However, YouTube's open platform allows registered users to upload content without a standardized peer-review process. Thus, we aimed to evaluate the quality and reliability of YouTube videos about OBR.

## Methods

A retrospective, cross-sectional analysis was conducted. Search terms were obtained from plasticsurgery.org and Google Trends. The first ten videos that appeared for each term were analyzed. Duplicates, non-English content, videos unrelated to OBR, or those lacking audio or text were excluded.

Video statistics and engagement metrics were collected. Videos were categorized by source type, content type, and OBR technique. The quality and reliability of videos were assessed using the DISCERN instrument, a validated questionnaire with a 5-point rating scale. Three independent researchers conducted video evaluations without cross-collaboration.

ANOVA and linear regression were performed using R. This was followed by Tukey's post-hoc test for pairwise comparisons. A two-sample *t*-test was conducted to assess the differences between video source categories. Data was presented as means ± standard deviations.

## Results

240 videos were identified, of which 172 were included for analysis. Videos that were duplicates (*n* = 50), unrelated (*n* = 16), non-English (*n* = 1), or part of larger playlists (*n* = 1) were excluded.

Five video source categories were identified: Health care administrations (HCA) (*n* = 5), physicians (*n* = 87), non-physician providers (*n* = 18), news organizations (*n* = 15), and patients (*n* = 47). News organizations had the most subscribers (3,870,480 ± 4,631,241), HCA had the longest videos (28.5 ± 34.5 min), and patients had the most likes (3749 ± 14,931) and comments (381 ± 1639). Physicians had the least views (42,764 ± 114,976).

Five content categories were identified: education (*n* = 66), job description (*n* = 11), live procedure (*n* = 25), news (*n* = 10), and patient experience (*n* = 60). Patient experiences had the most likes (3583 ± 13,843) and comments (340.8 ± 1471). Job descriptions had the fewest likes (18.9 ± 10.4) and views (2723 ± 1784). Live procedures were the oldest (15.9 ± 20.1 years) and had the longest duration (955.1 ± 1209 min).

Linear regression analysis demonstrated a positive correlation between video length and overall DISCERN score (*p* = 0.011). Views were negatively correlated with overall DISCERN scores (*p* = 0.001). Followers, video age, or comments did not correlate statistically with overall DISCERN scores.

Among the distinct sources, HCA accounts had the highest overall scores, while news organizations scored the lowest (3.6 ± 0.51 vs. 2.22 ± 0.60, *p* < 0.001) (Table 1). Among the distinct video categories, job description videos had the highest scores, while live procedures scored the lowest (3.37 ± 0.55 vs. 2.21 ± 0.92, *p* < 0.001). There was no statistically significant score difference between reconstructive approaches (*p* = 0.198).

**Table 1:** DISCERN scores for all videos included in this study.

	DISCERN Overall Score (Mean ± SD)	DISCERN Reliability Score (Mean ± SD)	DISCERN Quality Score (Mean ± SD)
Type of user account/source			
Health Care Administration	3.6 ± 0.51	3.3 ± 0.73	3.27 ± 0.37
Physician	2.98 ± 0.93	3.06 ± 0.90	3.10 ± 0.90
Non-physician Provider	2.81 ± 1.08	2.83 ± 0.91	3.04 ± 0.81
News Organization	2.22 ± 0.60	2.51 ± 0.76	2.07 ± 0.74
Patient	2.30 ± 0.82	2.48 ± 0.83	2.12 ± 0.81
*p-value*	*<0.001*	*<0.001*	*<0.001*
Type of video category			
Education	3.27 ± 0.85	3.29 ± 0.82	3.32 ± 0.84
Job Description	3.37 ± 0.55	3.42 ± 0.56	3.13 ± 0.82
Live Procedure	2.21 ± 0.92	2.34 ± 0.86	2.66 ± 0.86
News	2.33 ± 0.48	2.62 ± 0.70	2.03 ± 0.82
Patient Experience	2.29 ± 0.8	2.49 ± 0.82	2.19 ± 0.77
*p-value*	*<0.001*	*<0.001*	*<0.001*
Type of Surgery			
Autologous	2.71 ± 0.97	2.82 ± 0.99	2.80 ± 0.94
General	2.83 ± 0.93	2.87 ± 0.89	2.75 ± 0.98
Implant	2.65 ± 0.96	2.82 ± 0.85	2.68 ± 0.98
*p-value*	0.198	0.844	0.511

Data are presented as mean ± standard deviation.

Videos created by physicians, HCA, and other providers were labeled ``Academic,'' while videos from news organizations and patients were grouped as ``Non-academic.'' Compared to non-academic sources, academic sources had higher overall scores (2.98 ± 0.95 vs. 2.28 ± 0.77, *p* < 0.001), reliability scores (3.03 ± 0.90 vs. 2.49 ± 0.81, *p* < 0.001), and quality scores (3.10 ± 0.87 vs. 2.11 ± 0.79, *p* < 0.001) (Table 2).

**Table 2:** Videos characteristics and DISCERN scores based on academic vs. non-academic video source category.

	Academic Sources (*n* = 110)	Non-academic Sources (*n* = 62)	*p*-value
Video Characteristics			
No. videos	110	62	
No. account subscribers	234,681 ± 844,552	2229,788 ± 7055,175	*0.030*
Age of video (y)	4.71 ± 3.66	4.81 ± 3.10	0.853
Length of video (min)	11.7 ± 16.9	6.8 ± 6.0	*0.008*
No. likes	298.3 ± 760.9	3464 ± 13,629	*0.073*
No. views	69,978 ± 187,857	150,252 ± 528,751	0.252
No. comments	24.8 ± 72.0	331.6 ± 1447	0.100
DISCERN			
Overall score	2.98 ± 0.95	2.28 ± 0.77	*<0.001*
Reliability score	3.03 ± 0.90	2.49 ± 0.81	*<0.001*
Quality score	3.10 ± 0.87	2.11 ± 0.79	*<0.001*

Data are presented as mean ± standard deviation.

## Discussion

The findings of this study highlight the critical role that video source plays in determining the quality and reliability of YouTube content on OBR. Videos created by academic sources had increased reliability and quality. However, videos by physicians had the least views, and HCA only accounted for 3 % of all videos. This underscores the need for healthcare professionals to leverage YouTube more effectively by optimizing content for visibility and engagement.

Institutions and Physicians should consider collaborating with influencers, using targeted advertising, and optimizing content with appropriate tags to drive engagement. This can help ensure that patients seeking OBR information are exposed to reliable, high-quality sources.

Though this study is limited by its focus on the top ten videos for each search term and specific keywords, it offers valuable insight into the current landscape of OBR-related YouTube content and highlights opportunities for improving patient education.

## Conclusion

With patients increasingly turning to YouTube for OBR information, content quality is essential. Videos from academic sources, like healthcare administrations and physicians, are of higher quality and reliability but receive less engagement than non-academic sources, such as patients and news organizations. This gap in visibility for more reliable content underscores the need for healthcare professionals to actively create accessible, evidence-based videos to ensure patients receive accurate information.

## Conflict of interest

All authors report no conflicts of interest.
